# Comparative Effects of Dexamethasone and ASC Secretome in an Ex Vivo Osteoarthritis Co-Culture Model

**DOI:** 10.3390/biology15060493

**Published:** 2026-03-20

**Authors:** Elena Della Morte, Francesca Cadelano, Andrea Pasquini, Luigi Zagra, Alessandro Baj, Chiara Giannasi, Stefania Niada

**Affiliations:** 1 Laboratory of Biotechnological Applications, IRCCS Istituto Ortopedico Galeazzi, 20157 Milan, Italy; elena.dellamorte@grupposandonato.it (E.D.M.); francesca.cadelano@unimi.it (F.C.); alessandro.baj@unimi.it (A.B.); chiara.giannasi@unimi.it (C.G.); 2 Department of Biomedical, Surgical and Dental Sciences, University of Milan, 20129 Milan, Italy; 3 Hip Department, IRCCS Istituto Ortopedico Galeazzi, 20157 Milan, Italy; andrea.pasquini@unimi.it (A.P.); luigi.zagra@fastwebnet.it (L.Z.); 4 Università Vita-Salute San Raffaele, 20132 Milan, Italy

**Keywords:** osteoarthritis, mesenchymal stromal cells, secretome, conditioned medium, synovium–cartilage cross-talk, extracellular vesicles, matrix metalloproteinases, inflammation, ex vivo co-culture model, immunomodulation

## Abstract

Osteoarthritis (OA) is a multifactorial disease characterized by inflammation, extracellular matrix remodeling, and joint degeneration, and it still lacks disease-modifying treatments. Here, we applied an ex vivo OA model based on transwell co-cultures of cartilage and synovial membrane explants harvested from OA patients to compare the effects of adipose-derived stem/stromal cell (ASC) conditioned medium (CM) with dexamethasone (DEX), a clinically used corticosteroid. Explants were treated for 48 h with 100 nM DEX, CM derived from 5 × 10^5^ ASCs, or left untreated. Outcomes included gene and protein expression of key mediators, metalloprotease and aggrecanase activities, and nitric oxide release. DEX significantly reduced inflammatory markers (e.g., *PTGS*, *IL-1β*, and IDO) and *VEGF* expression in both tissues, while CM did not elicit consistent anti-inflammatory effects. Regarding matrix remodeling, both treatments reduced metalloprotease activity, with DEX modulating *MMP3* and *MMP13* expression in both tissues and CM reducing only *MMP3* expression in cartilage while presenting high levels of TIMP-1. These results confirm the robustness of the model, demonstrated by reproducible responses to DEX and its high-throughput potential, and underscore the need for mechanistic studies to optimize novel biotherapeutics.

## Simple Summary

Osteoarthritis is a common degenerative joint disease that still lacks effective treatments able to modify its progression. In this study, we used an experimental model that reproduces key features of the diseased joint by culturing cartilage and synovial membrane under controlled conditions. We compared the effects of dexamethasone, a widely used anti-inflammatory drug, with factors released by stem cells, collectively referred to as the secretome, and evaluated inflammation-related signals and the activity of enzymes involved in tissue remodeling. Dexamethasone effectively reduced inflammatory mediators in both cartilage and synovial membrane. The stem cell secretome showed more limited effects, mainly modulating matrix remodeling markers. These findings confirm the usefulness of this experimental model for comparing established and emerging therapies and support its application to investigate how new stem cell-based approaches may act on joint tissues.

## 1. Introduction

Osteoarthritis (OA) is a degenerative joint disease affecting 7.6% of the global population—about 595 million individuals worldwide [1]. Its prevalence increased by 48% between 1990 and 2019, and in 2019, OA ranked as the 15th leading cause of years lived with disability (YLDs), accounting for 2% of the global YLD burden [2]. OA is characterized by joint inflammation, fibrosis, extracellular matrix (ECM) degradation, neovascularization, and neural sensitization [3]. Clinically, these changes manifest as pain, reduced mobility, and loss of function [1].

The pathological process in OA is multifaceted and involves several joint tissues beyond cartilage, including the synovial membrane, and subchondral bone [4]. The synovial membrane plays a central role in maintaining joint homeostasis. Its intimal layer, composed of macrophages and fibroblast-like synoviocytes, and the subintimal layer, which houses blood vessels, lymphatic vessels, and infiltrating immune cells, together regulate lubrication, nutrient supply, immune surveillance, and ECM turnover [4,5]. In OA, the synovium exhibits increased infiltration of innate and adaptive immune cells, including macrophages, T cells, B cells, and plasma cells. Activated macrophages, in particular, are key contributors to synovial inflammation; their numbers rise with increasing synovitis severity. Pro-inflammatory cytokines released by these cells—such as IL-1β, TNF-α, and IL-6—propagate inflammatory responses and promote cartilage degradation. Neovascularization further facilitates the influx of immune cells, sustaining the chronic inflammatory state [1]. Notably, synovial inflammation often precedes detectable cartilage and bone alterations, underscoring the importance of the synovium–cartilage cross-talk in OA onset and progression [6].

Given the self-perpetuating inflammatory loop that characterizes OA, multiple therapeutic strategies aim to interrupt this cycle. Among anti-inflammatory approaches, intra-articular glucocorticoids have been widely used for the treatment of symptomatic OA. Glucocorticoids exert their effects by suppressing inflammation through inhibition of pro-inflammatory cytokines, prostaglandins, and matrix-degrading enzymes. In OA, where chronic low-grade inflammation and catabolic signaling drive cartilage degradation and synovial activation, glucocorticoid-mediated transcriptional repression can temporarily attenuate inflammatory and matrix-degrading pathways, thereby providing short-term symptomatic relief. However, prolonged modulation of gene expression may adversely affect cartilage homeostasis and tissue integrity, with accumulating evidence suggesting potential cartilage damage and even accelerated disease progression [7,8,9].

In this context, mesenchymal stem/stromal cells (MSCs) have emerged as a promising therapeutic alternative due to their regenerative and immunomodulatory properties. Increasing evidence indicates that many of their therapeutic effects are mediated not by the cells themselves but by their secreted bioactive factors—collectively known as the MSC secretome or conditioned medium (CM). The secretome includes cytokines, chemokines, growth factors, lipids, and extracellular vesicles containing functional miRNAs and proteins. Secretome-based, cell-free approaches offer several advantages over cellular therapies, including reduced immunogenicity, improved safety, and easier handling and storage. Importantly, the MSC secretome has demonstrated strong immunomodulatory potential, making it particularly relevant for conditions, such as OA, where dysregulated immune activity plays a pivotal role [10].

Given the central involvement of immune cells and the dynamic interplay between synovium and cartilage in OA progression, it is important to evaluate therapeutic strategies in disease-relevant models that capture this complexity. For these reasons, we compared the secretome of MSCs derived from adipose tissue and dexamethasone, a widely used glucocorticoid, using an optimized ex vivo model involving both cartilage explants and synovial membrane explants from the same donor. This model provides a physiologically relevant inflammatory environment, preserves the synovium–cartilage cross-talk, and better reflects the immune contributions that are crucial in driving OA pathology.

## 2. Materials and Methods

### 2.1. Cell and Tissue Cultures

Cells and tissue were isolated from waste tissues collected at IRCCS Ospedale Galeazzi-Sant’Ambrogio under the TENET protocol, approved by the Ethics Committee of IRCCS Ospedale San Raffaele (approval number 38/int/2022). Specifically, adipose-derived stem/stromal cells (ASCs) were obtained from subcutaneous adipose tissue of 8 patients (4 males and 4 females, aged 69 ± 12 years) undergoing total hip arthroplasty (Supplementary Table S1). Tissues were minced using a scalpel and digested with 0.75 mg/mL collagenase type I (Worthington Biochemical Corporation, Lakewood, NJ, USA) at 37 °C for 30 min. The resulting cell suspensions were filtered through a 100 µm cell strainer (Corning Incorporated, Corning, NY, USA). Cells were plated at a density of 10^4^ cells/cm^2^ in a standard culture medium composed of high-glucose Dulbecco’s Modified Eagle’s Medium (DMEM) supplemented with 10% fetal bovine serum (FBS HyClone, Euroclone, Pero, Italy), 2 mmol/L glutamine, 50 µg/mL streptomycin, and 50 U/mL penicillin.

Synovial membrane and cartilage explants were obtained from 7 patients (4 males and 3 females, aged 61 ± 11 years, Supplementary Table S1). Membrane sections were isolated, excluding cauterization sites and infiltrated adipose tissue, and then cut into pieces approximately 2–3 mm in size. Cartilage was excised from macroscopically preserved regions of the femoral head, avoiding arthritic lesions or calcified areas. Subsequently, 4 mm biopsy punch explants were obtained. Following extraction, samples were washed with phosphate-buffered saline (PBS) and cultured in a medium composed of DMEM *w*/*o* phenol red, 1% FBS, 2 mM L-Glutamine, 150 U/mL Penicillin, 150 µg/mL Streptomycin, and 2.5 µg/mL Amphotericin-β for 1 day. Next, synovial membrane and cartilage explants (60 mg each) were co-cultured in Corning^®^ 12 mm Transwell^®^ with 0.4 µm Pore Polycarbonate Membrane Inserts, allowing physical separation while permitting exchange of soluble factors. Cartilage explants were allocated in the lower compartment of the plate, and synovial explants were positioned in the insert. A total of 750 µL of medium was added to each compartment. The following day, treatment day, the synovial membrane compartment was treated for 48 h as follows:

1. Control group (CTR);
2. 100 nM dexamethasone (DEX);
3. CM-derived from 5 × 10^5^ ASCs (CM).

The dexamethasone dose was selected according to Chan et al. [11], in which the same dose was shown to exert anti-inflammatory and chondroprotective effects in the cartilage/synovial explant model. The CM dose was chosen based on our previous investigations in 2D chondrocyte cultures [12], cartilage and synovial explants [13], and a compartmentalized osteochondral model [14], and it is also consistent with the study by Van Buul et al. [15]. Neither treatment produced significant changes in the viability of the co-culture (Supplementary Figure S1). Supernatants were collected and stored at −20 °C until use. At the endpoint, explants were washed with PBS and preserved at −80 °C.

### 2.2. CM Production

Conditioned medium was generated using cells at the 4th–5th culture passage, following established laboratory protocol [16]. Briefly, cells were washed twice with PBS, followed by a 5 min wash with standard medium lacking FBS and phenol red. ASCs were then cultured for three days under serum-starvation conditions. After this period, the culture medium was collected and centrifuged at 2500× *g* for 10 min at 4 °C to remove cellular debris. The supernatant was concentrated using Amicon Ultra-15 centrifugal filter units (3 kDa cut-off; Merck Millipore, Burlington, MA, USA), aliquoted, and stored at −80 °C. In parallel, cells were detached and counted to correlate the number of donor cells with the volume of CM obtained.

### 2.3. CM Characterization

For the experimental treatments, the CM were pooled to minimize inter-donor variability, ensuring a consistent and standardized approach in the study.

#### 2.3.1. Protein Quantification

Total protein concentration in CM was measured using the Bradford assay (Bio-Rad, Milan, Italy), according to standard protocols.

#### 2.3.2. Nanoparticle Tracking Analysis (NTA)

Aliquots of CM derived from 4 × 10^5^ cells were diluted to a final volume of 800 µL with 0.22 µm triple-filtered PBS and analyzed using the NanoSight NS3000 (Malvern Panalytical, Salisbury, UK). For each sample, three 1 min videos were recorded. Measurements adhered to quality criteria: 20–120 particles/frame, 10^6^–4 × 10^9^ particles/mL, and >20% valid tracks. Videos were analyzed with NanoSight NTA software (version NTA 3.4 Build 3.4.003).

#### 2.3.3. Luminex Discovery Assay

Cytokine and growth factor levels in CM were quantified using the Human Premixed Multi-Analyte Kit LXSAHM (R&D Systems, Minneapolis, MN, USA). The panel included CCL2, CCL3, CXCL1, HGF, G-CSF, M-CSF, TIMP-1, IL-RA, IL-10, VEGFA, IL-8, IL-4, OPG, and IFNG. Samples were diluted 1:2–1:500, tested in duplicate, and analyzed using a Bio-Plex Multiplex System (Bio-Rad, Milan, Italy). Data were processed with MAGPIX PONENT 4.2 software (Luminex Corporation, Austin, TX, USA).

#### 2.3.4. ELISA (TGF-β1 and IL-6)

TGF-β1 and IL-6 were quantified in undiluted and 1:500 diluted CM with a Human/Mouse/Rat/Porcine/Canine TGF-beta 1 ELISA Kit—Quantikine (R&D Systems, Minneapolis, MN, USA) and a Human IL-6 ELISA kit (Thermo Fisher Scientific, Waltham, MA, USA) following kit instructions.

#### 2.3.5. Cytofluorimetry

Prior to cytofluorimetric analysis, the CM pool was diluted 1:10 in 0.22 μm triple-filtered PBS, stained with 100 nM CFSE-DA (carboxyfluorescein diacetate succinimidyl ester, Biotium, Fremont, CA, USA) for 1 h at 37 °C, and analyzed without further washing. Data acquisition was performed using a CytoFLEX flow cytometer (Beckman Coulter, Brea, CA, USA). Instrument calibration was set using a Megamix-Plus SSC and FSC mix (Biocytex, Marseille, France), a reference bead mixture containing FITC fluorescent spheres of varying sizes (100 nm, 160 nm, 200 nm, 240 nm, 300 nm, 500 nm, and 900 nm). CFSE-positive (CFSE^+^) CM was analyzed in the Violet SSC-H and FITC-H channels, with event regions defined according to standardization bead coordinates. CFSE^+^ samples were then aliquoted and incubated for 30 min at 4 °C in the dark with APC-conjugated antibodies against CD9, CD63, and CD81 (BioLegend, San Diego, CA, USA; 1:20 dilution). Samples were diluted to a final volume of 300 μL in 0.22 μm triple-filtered PBS, and 50,000 events were acquired at a medium flow rate. DMEM-diluted antibodies and unlabeled samples were used as controls.

### 2.4. Gene and Protein Expression

Tissue explants were subjected to RNA and protein extraction by the TRIzol-Chloroform extraction method, utilizing PreCellys Mini Tubes (Bertin Technologies, Montigny-le-Bretonneux, France). The resulting RNAs were then retrotranscribed using the HighCapacity cDNA Reverse Transcription kit (Applied Biosystems, Foster City, CA, USA), following the manufacturer’s protocol. Subsequently, the expression levels of different genes were quantified through reverse transcription quantitative polymerase chain reaction (RT-qPCR) employing TaqMan technology using the following probes: *PTGS2* (hs00153133_m1), *MMP3* (hs00968305_m1), *MMP13* (hs00 233992_m1), *SOX9* (hs00165814_m1), *COL2A1* (hs01060345_m1), *IL-1β* (Hs01555410_m1), *TIMP1* (Hs99999139_m1), *ACAN* (hs00153936_m1), *COL10A1* (hs00166657_m1), *RUNX2* (hs00231692_m1), *VEGFA* (hs00900055_m1), *FN1* (Hs01549976_m1), *CTGF* (Hs00170014_m1), and *TBP* (Hs00427620_m1) (Thermo Fisher Scientific, Waltham, MA, USA). The RT-qPCR assays were conducted on the QuantStudio Real-Time PCR systems (Thermo Fisher Scientific, Waltham, MA, USA). Data normalization was performed using *TBP* as a reference, and relative quantification was determined employing the 2^−ΔΔCT^ method.

Protein concentrations were determined using the BCA assay (Thermo Fisher Scientific, Waltham, MA, USA). Subsequently, 10 µg of total proteins per sample were loaded and separated on a 10% SDS-PAGE gel. Western blotting was performed following standard protocols. Primary antibodies—rabbit anti-COX2 (#12282, Cell Signaling, Danvers, MA, USA; 1:1000 dilution), rabbit anti-IDO (#86630, Cell Signaling, Danvers, MA, USA; 1:1000 dilution), rabbit anti-COL2A1 (ab34712, Abcam, Cambridge, UK; 1:2000 dilution), and anti-GAPDH (sc-47724, Santa Cruz Biotechnology, Santa Cruz, CA, USA; 1:750 dilution)—were incubated overnight at 4 °C. Specific signals were detected using appropriate horseradish peroxidase-conjugated secondary antibodies (Thermo Fisher Scientific, Waltham, MA, USA; 1:3000 dilution) and visualized with ECL Westar Supernova (Cyanagen, Bologna, Italy). Signal acquisition was carried out using a ChemiDoc Imaging System, and densitometric analysis was performed using Image Lab Software version 6.1 (Bio-Rad, Milan, Italy).

### 2.5. Evaluation of Matrix Metalloproteinases (MMP) and Aggrecanase-1 Activity

The activity of MMPs and Aggrecanases-1 was assessed using the SensoLyte 520 Generic MMP Activity Kit and the SensoLyte^®^ 520 Aggrecanase-1 Assay Kit Fluorimetric, respectively (AnaSpec, Fremont, CA, USA). For MMPs, pro-enzymes were activated by incubating undiluted samples with 1 mM 4-aminophenyl mercuric acetate for 40 min at 37 °C. Both assays were then performed with their respective substrates for 45 min, and fluorescence signals (excitation λ = 490 nm, emission λ = 520 nm) were measured using a Wallac Victor II microplate reader (Perkin Elmer, Milan, Italy).

### 2.6. Measurement of Released Sulphated Glycosaminoglycans (sGAGs)

The release of sGAGs into culture supernatants was quantified using the dimethyl-methylene blue (DMMB) method (Sigma Aldrich, St. Louis, MO, USA). A standard curve was generated using chondroitin sulfate with concentrations ranging from 1.6 to 25 μg/mL. In the assay, 50 μL of standards or samples were combined with 200 μL of DMMB reagent. The absorbance at 500 nm was promptly measured using a Wallac Victor II microplate reader.

### 2.7. NO Assay

The quantification of nitric oxide (NO) levels was performed utilizing the Nitric Oxide Assay kit from Abcam (ab272517, Abcam, Cambridge, UK). Undiluted samples underwent processing in accordance with the provided protocol. Samples were deproteinized, incubated for 10 min at 60 °C, and finally, the oxidized NO content was quantified by measuring the optical density at 540 nm with a Wallac Victor II spectrophotometer. Extrapolation of NO levels was derived from a nitrite standard curve (0–200 mM).

### 2.8. Statistics

All data were analyzed using GraphPad Prism (version 8.3.0, GraphPad Software, San Diego, CA, USA). Statistical analysis was performed using one-way analysis of variance (ANOVA) to compare mean values among multiple groups. Data are presented as mean ± standard error of the mean (SEM). A *p*-value ≤ 0.05 was considered statistically significant.

## 3. Results

### 3.1. Secretome Characterization

**Figure 1:**
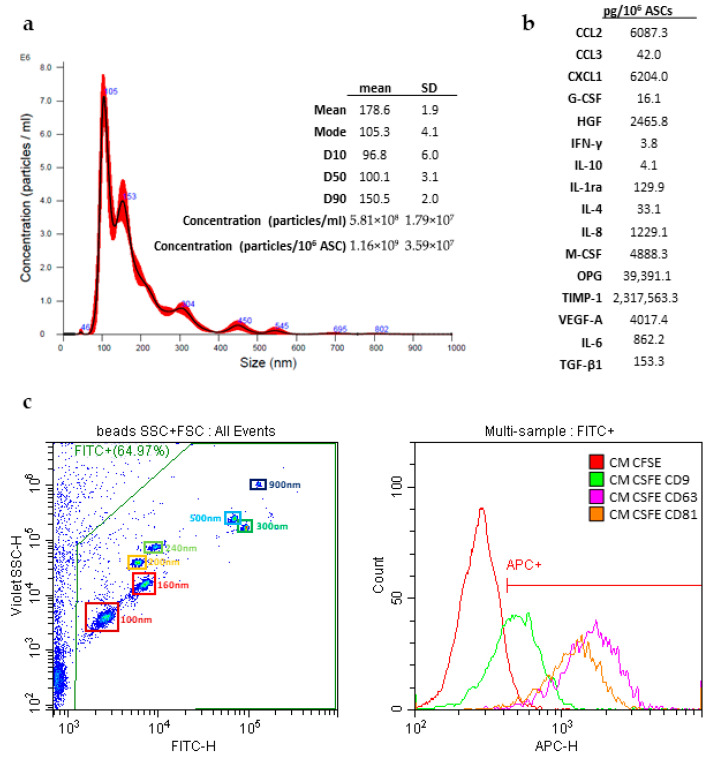
(**a**) Nanoparticle tracking analysis (NTA) of CM pool and a table showing the dimensional parameters of the sample expressed as mean ± SD of 3 technical replicates. (**b**) Levels (pg per 10^6^ ASC) of selected proteins in the CM pool. (**c**) Calibration for dimensional gating and debris exclusion was made using FITC^+^ beads with known sizes (corresponding diameters of 100, 160, 200, 240, 300, 500, and 900 nm). CM pool CFSE^+^ vesicles that showed positivity for CD9, CD63, and CD81.

The pooled CMs were characterized in terms of both particle and protein content. Nanoparticle tracking analysis (NTA) revealed a particle concentration of 1.16 × 10^9^ particles per million ASCs, with a mean diameter of 178.6 ± 1.9 nm and a mode size of 105.3 ± 4.1 nm. The D90 (diameter below which 90% of particles fall) was 150 nm, while the D10 (diameter below which 10% of particles fall) was 97 nm, indicating a relatively narrow size distribution, with most particles concentrated within this range (Figure 1a).

The extracellular vesicles (EVs) present in the CM were positive for classical vesicular markers, the tetraspanins CD9 (56.5%), CD63 (90.8%), and CD81 (88.8%), confirming their vesicular nature (Figure 1c).

In terms of protein content, the pooled CM protein concentration was 0.84 µg/µL. Additionally, the release of specific factors involved in immune cell recruitment, regulation, and tissue regeneration was evaluated using a Luminex assay. These factors included CCL2, CCL3, CXCL1, G-CSF, HGF, IFN-γ, IL-10, IL1RA, IL-4, IL-8, M-CSF, OPG, TIMP1, VEGFA, IL-6, and TGF-β. Their concentrations were normalized per million ASCs, and the results are shown in Figure 1b. Overall, these data indicate that the ASC secretome contains both vesicular and soluble components with potential immunomodulatory and regenerative activity.

### 3.2. Effects on Inflammation and Angiogenesis on Synovial and Cartilage Tissues

**Figure 2:**
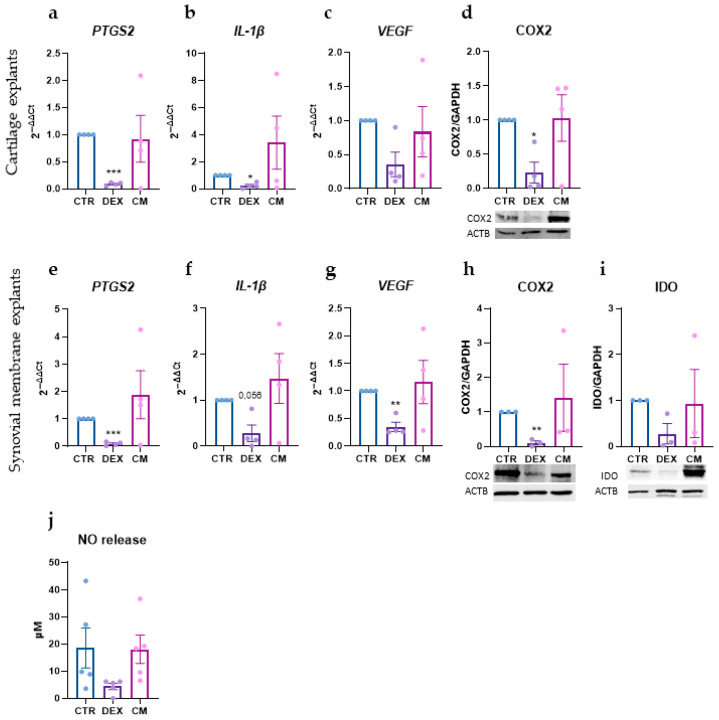
(**a**–**c**) Gene expression of *PTGS2* (**a**), *IL-1B* (**b**), and *VEGFA* (**c**) in cartilage explants (CEs) after the treatments. Data are expressed as 2^−ΔΔCt^ (TBP was used as a housekeeping gene) (**d**) COX2 expression in CEs after the treatments and a representative immunoblot (below). Data were normalized to GAPDH expression and expressed as relative values (CTR = 1). (**e**–**g**) Gene expression of *PTGS2* (**e**), *IL-1β* (**f**), and *VEGFA* (**g**) in synovial membrane explants after the treatments. Data are expressed as 2^−ΔΔCt^ (TBP was used as a housekeeping gene) (**h**,**i**) COX2 (**h**) and IDO (**i**) expression in synovial membrane explants after the treatments and representative immunoblots (below). Data were normalized to GAPDH expression and expressed as relative values (CTR = 1). (**j**) Nitric oxide (NO) release (µM) in supernatants. Data are expressed as mean ± SEM of *n* = 4–5 independent experiments. Significance versus CTR is shown as * *p* < 0.05, ** *p* < 0.01, and *** *p* < 0.001.

The impact of treatments on inflammatory and angiogenic markers was evaluated in the two-tissue model. In both cartilage explants and synovial membrane, DEX effectively reduced the gene expression of inflammatory markers, specifically *PTGS2* and *IL1B*, which was further confirmed by decreased COX2 protein levels (Figure 2a,b,d–f,h and File S1).

In the synovial membrane, DEX also reduced IDO protein expression (Figure 2i). Furthermore, DEX decreased *VEGF* gene expression in synovial membrane explants and had a modest effect in the cartilage equivalent (Figure 2c,g). In contrast, the CM treatment did not show clear or consistent effects in these contexts (Figure 2).

The effects of the treatments were also reflected in the secretion of inflammatory mediators: DEX reduced the release of nitric oxide (NO), consistent with its anti-inflammatory activity, while CM induced no changes (Figure 2j).

### 3.3. Effects of Treatments on Matrix Remodeling and Fibrosis

The effects of the treatments on matrix remodeling and fibrosis were evaluated after two days of exposure.

**Figure 3:**
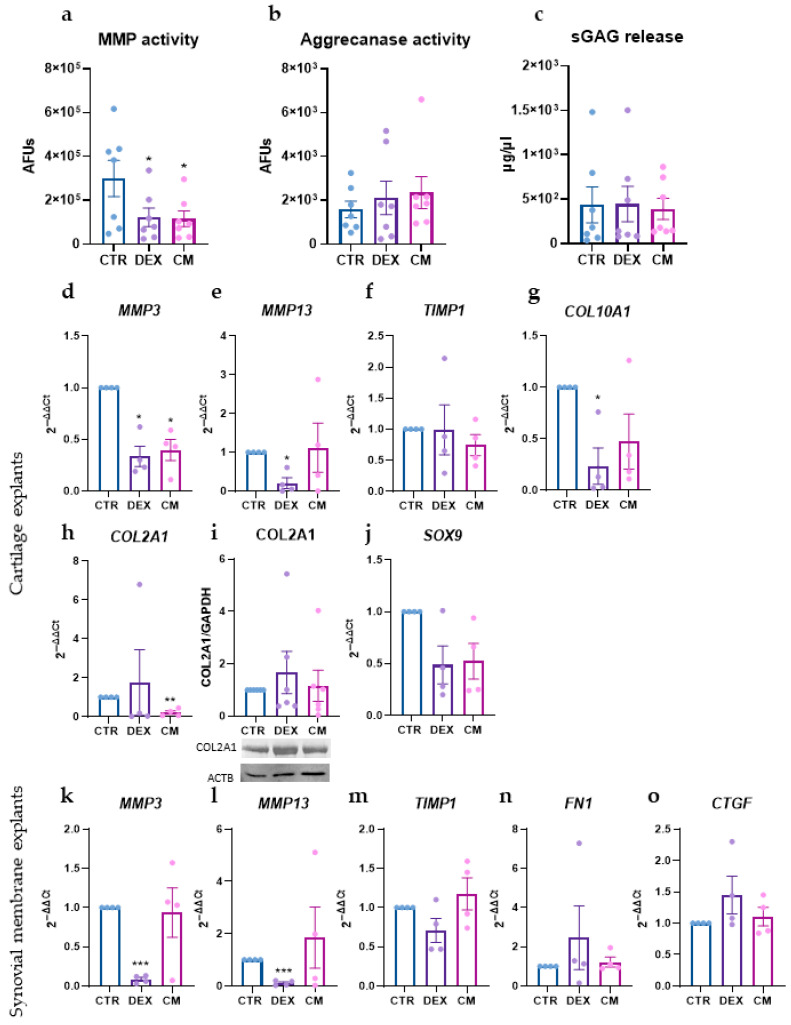
(**a**,**b**) MMP (**a**) and aggrecanase 1 (**b**) activity in supernatants. Data are expressed as arbitrary fluorescence units (AFUs). (**c**) sGAG release (µg/µL) in supernatants. (**d**–**j**) Gene expression of *MMP3* (**d**), *MMP13* (**e**), *TIMP1* (**f**), *COL10A1* (**g**), *COL2A1* (**h**), and *SOX9* (**j**) in cartilage explants after the treatments. Data are expressed as 2^−ΔΔCt^ (TBP was used as a housekeeping gene) (**i**) *COL2A1* expression in CE after the treatments and a representative immunoblot (below). Data were normalized to GAPDH expression and expressed as relative values (CTR = 1). (**k**–**o**) Gene expression of MMP3 (**k**), MMP13 (**l**), TIMP1 (**m**), FN1 (**n**), and CTGF (**o**) in synovial membrane explants after the treatments. Data are expressed as 2^−ΔΔCt^ (TBP was used as a housekeeping gene). Data are expressed as mean ± SEM of *n* = 4–7 independent experiments. Significance versus CTR is shown as * *p* < 0.05, ** *p* < 0.01, and *** *p* < 0.001.

The impact on matrix-degrading enzymes was assessed first. Both treatments significantly reduced MMP activity, while the activity of aggrecanase 1 remained unchanged (Figure 3a,b). DEX significantly reduced the gene expression of the two most representative MMPs in cartilage and synovial membrane, namely, *MMP3* and *MMP13*, whereas CM only reduced *MMP3* expression in cartilage (Figure 3d,e,k,l). The expression of *TIMP1*, a natural inhibitor of MMPs, was not significantly affected by either treatment (Figure 3f,m). This data suggests that DEX primarily acts by reducing MMP expression, whereas CM functions downstream by buffering MMP activity through TIMPs (see elevated TIMP-1 level in Figure 1b and [12,14,17]).

Analysis of genes involved in matrix composition and synthesis revealed that DEX was able to reduce the expression of *COL10A1* (Figure 3g), a type of collagen typically expressed by hypertrophic chondrocytes, whereas CM reduced *COL2A1* expression (Figure 3h), although this reduction was not confirmed at the protein level (Figure 3i and File S1). Both treatments also induced a variable reduction in *SOX9* expression (Figure 3j). Differently, neither treatment affected the release of glycosaminoglycans (GAGs) nor other genes involved in matrix composition (fibronectin 1, FN1) or matrix remodeling (connective tissue growth factor, CTGF) (Figure 3c,n,o).

## 4. Discussion

Osteoarthritis (OA) is a debilitating joint disease affecting millions of people worldwide and remains an area of high unmet clinical need, as current treatments are largely symptomatic and do not effectively halt disease progression. In this context, the secretome or conditioned medium (CM) derived from mesenchymal stem/stromal cells (MSCs), either in its complete form or as the isolated extracellular vesicle fraction, has emerged as a promising therapeutic strategy, with potential applications in inflammatory and autoimmune diseases as well as orthopedic conditions, including OA and OA-associated pain [18,19,20]. These novel approaches represent an alternative to traditionally used anti-inflammatory drugs such as corticosteroids. Among corticosteroids, dexamethasone (DEX) is a potent glucocorticoid widely used in the clinical management of OA for short-term pain relief [21] and has been shown to mitigate the deleterious effects induced by mechanical injury and pro-inflammatory cytokines in human cartilage explants in vitro [22]. DEX and CM exert their effects through fundamentally different mechanisms. Glucocorticoids primarily act through modulation of gene expression via activation of the glucocorticoid receptor (GR). In the canonical pathway, ligand-bound GR functions as a transcription factor by binding to glucocorticoid response elements to regulate target gene expression. In the non-canonical pathway, GR interacts with and represses key pro-inflammatory transcription factors, such as NF-κB and AP-1 [23]. By contrast, the secretome is a highly complex and heterogeneous mixture composed of thousands of bioactive factors that exert their biological effects at multiple levels through diverse and complementary mechanisms, ranging from post-transcriptional regulation of gene expression via miRNAs to downstream effects mediated by extracellular modulators [24,25].

To investigate the therapeutic potential of MSC-derived CM in a disease-relevant setting, we compared its effects with those of DEX using an ex vivo cartilage–synovium co-culture model. This co-culture system, previously described by van Buul et al. [15] and Chan et al. [11], consists of cartilage and synovial membrane explants obtained from the same OA patient and maintained in a transwell configuration. This setup allows the exchange of soluble factors while preserving tissue-specific characteristics, making it particularly suitable for evaluating therapeutic strategies, such as MSC-CM, whose effects depend on complex tissue–tissue communication rather than isolated cellular responses. In both our study and previous reports, tissues were harvested from osteoarthritic donors, which represents a key strength of the model. Indeed, as demonstrated by Chan et al., the presence of OA synovium increases cartilage metalloproteinase expression (e.g., *MMP1* and *MMP13*) and inflammatory mediators, such as *PTGS2*, even in the absence of exogenous cytokine stimulation [11]. In the present study, pooled CM was used to minimize inter-donor variability. The CM pool exhibited the characteristic features of MSC secretome previously described by us and others, being enriched in extracellular vesicles, growth factors, and immunomodulatory and tissue-remodeling mediators [17,18,26]. Following the characterization of CM composition, in vitro treatments were performed. The first major observation was that DEX markedly reduced all inflammatory markers analyzed (namely, COX2, *IL-1β*, and IDO expression), reflecting the well-established capacity of corticosteroids to suppress the expression of multiple inflammatory genes, including cytokines, chemokines, and inflammation-associated enzymes [27]. In addition to its anti-inflammatory effects, DEX also reduced the expression of the angiogenic factor *VEGF* in the synovial membrane, highlighting its ability to interfere with inflammation-driven angiogenesis within this compartment. This observation is consistent with previous studies showing corticosteroid-mediated reduction in VEGF expression in various cell types, including vascular smooth muscle cells [28] and hemangioma-derived stem cells [29].

In contrast, ASC-CM did not significantly affect inflammatory or angiogenic markers under the experimental conditions used. This finding is consistent with previous evidence indicating that ASCs often require priming with inflammatory cytokines or hypoxia—both hallmarks of the OA joint microenvironment—to fully exert their immunomodulatory properties [30,31]. For example, Cifù et al. demonstrated that only the secretome derived from ASCs primed with synovial fluid from OA patients, but not the one derived from naïve ASCs, was able to suppress T cell proliferation and promote regulatory T cell expansion [32]. These findings suggest that the lack of immunomodulatory activity under standard culture conditions does not preclude the intrinsic anti-inflammatory potential of ASCs; rather, it highlights the importance of environmental cues in licensing their functional phenotype. Accordingly, several priming strategies have been explored to enhance ASC therapeutic efficacy, including exposure to pro-inflammatory cytokines, hypoxic preconditioning, and three-dimensional culture systems, all of which have been shown to modulate the composition and biological activity of the secretome [30,33,34].

Despite the different effects on inflammatory markers, both DEX and ASC-CM significantly reduced MMP activity. The effect of ASC-CM confirms our previous observations across multiple experimental models, including 2D cultures of articular chondrocytes [12], cartilage explants [13], and osteochondral explants [35], while the inhibitory effect of DEX is consistent with previous findings by Chan et al. [11]. In the case of DEX, reduced MMP activity can be primarily attributed to decreased *MMP* expression. Conversely, the effect of ASC-CM is likely mainly driven by the high abundance/markedly elevated levels of tissue inhibitors of metalloproteinases (TIMPs), which we previously identified as the principal mechanism responsible for MMP activity reduction [12,13,35]. TIMPs exert their function through direct enzymatic inhibition of MMPs, forming complexes with active MMPs and thereby blocking their proteolytic activity. In the present study, the observed reduction in MMP activity may, therefore, mainly reflect this post-translational regulatory mechanism, although it could also be partially supported by the concomitant decrease in *MMP3* expression. Despite the reduction in MMP activity, neither treatment significantly influenced glycosaminoglycan (GAG) loss. This may be due to the relatively short duration of the experimental setting (48 h) and is consistent with previous observations [11,13]. Notably, we confirmed a CM-induced reduction in the expression of key cartilage-related genes involved in chondrocyte differentiation and extracellular matrix maintenance, as previously reported [13,14]. Importantly, ASC-CM did not appear to induce cartilage hypertrophy, as collagen type X expression was also reduced, in line with earlier findings [12]. This observation warrants further investigation, particularly in light of the growing interest in orthobiological approaches for the treatment of osteoarthritis. Studies on matrix remodeling should be conducted using longer culture protocols. In the present study, a short-term culture model was employed. Although this approach is suitable for evaluating acute biochemical responses, such as changes in inflammatory markers [11,15], it does not allow comprehensive assessment of long-term matrix remodeling processes, which require extended culture periods ranging from days to weeks [36,37]. Extending the culture duration would also strengthen the comparison with dexamethasone. Although dexamethasone exerts anti-inflammatory effects and provides short-term symptomatic relief, prolonged exposure has been associated with adverse effects, including reduced cartilage volume and thickness, chondrocyte senescence and apoptosis, and impaired proteoglycan synthesis [38,39]. Therefore, longer culture durations, potentially combined with the addition of inflammatory cytokines to sustain a stimulated microenvironment, should be considered in future studies to better elucidate these effects and further validate the observed responses.

## 5. Conclusions

Overall, these findings confirm that the cartilage–synovium co-culture model is highly responsive to pharmacological modulation and is capable of capturing treatment-dependent differences in both inflammatory responses and matrix remodeling processes that are relevant to OA pathology. In this experimental setting, CM did not exert major effects, apart from a clear inhibition of MMP activity. With this study, we do not intend to downplay the therapeutic potential of MSCs and their secretome, which we ourselves have been investigating for several years as modulators of OA, achieving very encouraging results, including in vivo evidence of pain reduction and neuroinflammation [19,40]. However, it should be acknowledged that these orthobiological products still require further optimization. These products are rich in biologically active factors and are highly tunable yet also variable, depending on donor cell characteristics and priming conditions. Priming/licensing can be achieved through treatment with pro- or anti-inflammatory cytokines, different culture conditions such as hypoxia or three-dimensional cultures, or combinations of these approaches. In addition, the identification of the optimal dose is an important aspect and could represent a meaningful extension of the present study. Selecting specific subcomponents of the CM or removing potentially harmful factors from the CM (e.g., cytokines that activate the immune system) may also allow safer dose escalation while preserving or enhancing therapeutic efficacy. Moreover, it should be noted that MSCs differ in efficacy depending on their tissue of origin; therefore, changing the donor cell source may represent another potentially effective strategy. Finally, although the pooling strategy is useful for performing a robust overall assessment of the biological effects of the medium while reducing inter-donor variability, it may also mask donor-specific differences and limit correlations between individual CM characteristics and biological potency. Therefore, the analysis of single patient-derived CM should also be performed to enable patient stratification and to identify CM preparations that exert distinct biological effects. Therefore, all these aspects must be carefully investigated in order to identify the most appropriate product for the specific pathological context of interest.

## Data Availability

The datasets supporting the conclusions of this article is available in the Zenodo repository (10.5281/zenodo.19063782).

## Supplementary Materials

The following supporting information can be downloaded at: https://www.mdpi.com/article/10.3390/biology15060493/s1, File S1. WB figures. Figure S1. Explants viability at day 2 expressed as arbitrary fluorescence units (AFUs) and normalized to day 0. Table S1: Donors information.

## Abbreviations

The following abbreviations are used in this manuscript:

ACAN	Aggrecan
ANOVA	Analysis of Variance
APC	Allophycocyanin
ASCs	Adipose-Derived Stem/Stromal Cells
BCA assay	Bicinchoninic Acid Assay
CCL	C-C Motif Chemokine Ligand
CD9/CD63/CD81	Cluster of Differentiation 9/63/81
CFSE	Carboxyfluorescein Succinimidyl Ester
CM	Conditioned Medium
COL10A1	Collagen Type X Alpha 1 Chain
COL2A1	Collagen Type II Alpha 1 Chain
COX-2	Cyclooxygenase-2
CTGF	Connective Tissue Growth Factor
CTR	Control
CXCL	C-X-C Motif Chemokine Ligand
DEX	Dexamethasone
DMEM	Dulbecco’s Modified Eagle’s Medium
DMMB	Dimethylmethylene Blue
ECL	Enhanced Chemiluminescence
ECM	Extracellular Matrix
ELISA	Enzyme-Linked Immunosorbent Assay
EVs	Extracellular Vesicles
FBS	Fetal Bovine Serum
FITC	Fluorescein Isothiocyanate
FN1	Fibronectin 1
GAPDH	Glyceraldehyde-3-Phosphate Dehydrogenase
G-CSF	Granulocyte Colony-Stimulating Factor
HGF	Hepatocyte Growth Factor
IDO	Indoleamine 2,3-Dioxygenase
IFN-γ/IFNG	Interferon Gamma
IL	Interleukin
IL-10	Interleukin-10
IL1RA/IL-RA	Interleukin-1 Receptor Antagonist
IL-1β	Interleukin-1 Beta
IL-4	Interleukin-4
IL-6	Interleukin-6
IL-8	Interleukin-8
kDa	Kilodalton
M-CSF	Macrophage Colony-Stimulating Factor
MMP	Matrix Metalloproteinase
MMP13	Matrix Metalloproteinase-13
MMP3	Matrix Metalloproteinase-3
MSCs	Mesenchymal Stem/Stromal Cells
NO	Nitric Oxide
NTA	Nanoparticle Tracking Analysis
OA	Osteoarthritis
OPG	Osteoprotegerin
PBS	Phosphate-Buffered Saline
PCR	Polymerase Chain Reaction
PTGS2	Prostaglandin–Endoperoxide Synthase 2
RT-qPCR	Reverse Transcription Quantitative Polymerase Chain Reaction
RUNX2	Runt-Related Transcription Factor 2
SDS-PAGE	Sodium Dodecyl Sulfate Polyacrylamide Gel Electrophoresis
SEM	Standard Error of the Mean
SM	Synovial Membrane
SOX9	SRY-Box Transcription Factor 9
SSC/FSC	Side Scatter/Forward Scatter
TBP	TATA-Box Binding Protein
TGF-β1	Transforming Growth Factor Beta 1
TIMP/TIMP1	Tissue Inhibitor of Metalloproteinases
TNF-α	Tumor Necrosis Factor Alpha
VEGF/VEGFA	Vascular Endothelial Growth Factor A
YLDs	Years Lived with Disability
