# Supplementary material for: Comparative Effects of Dexamethasone and ASC Secretome in an Ex Vivo Osteoarthritis Co-Culture Model
Source: Biology (Basel). 2026 Mar 20;15(6):493. doi: 10.3390/biology15060493 (PMC13023977; doi:10.3390/biology15060493)
Supplement: Supplementary file 1 [file biology-15-00493-s001.zip › biology-4175071-Figure S1.pdf]

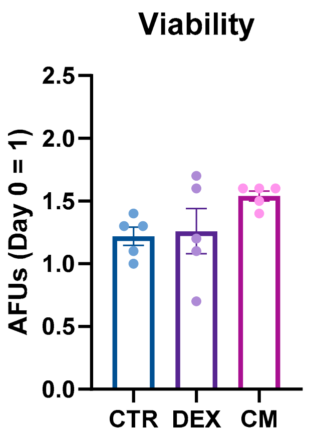

Supplementary Figure S1. Explants viability at day 2 expressed as arbitrary fluorescence units (AFUs) and normalized to day 0. Data are expressed as mean ± SEM of n = 5 independent experiments.

Tissues viability was evaluated prior to treatment (T0) and at the experimental endpoint (day 2) using the AlamarBlue assay (Thermo Fisher Scientific, Waltham, MA, USA). Explants were incubated with a 10% (v/v) AlamarBlue solution prepared in culture medium for 2 hours at 37°C. Following incubation, 100 μL aliquots from each well were collected in triplicate and transferred to black-bottom 96-well plates. Fluorescence was measured using a Wallac Victor II microplate reader (PerkinElmer, Milan, Italy) with excitation at 530 nm and emission at 590 nm.
