# Supplementary material for: Comparative Effects of Dexamethasone and ASC Secretome in an Ex Vivo Osteoarthritis Co-Culture Model
Source: Biology (Basel). 2026 Mar 20;15(6):493. doi: 10.3390/biology15060493 (PMC13023977; doi:10.3390/biology15060493)

Fig2d and Fig3i

Exp3

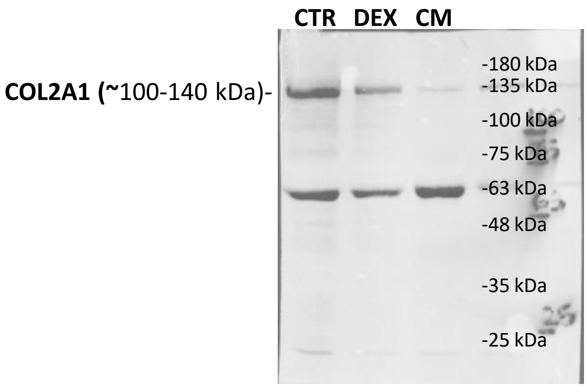

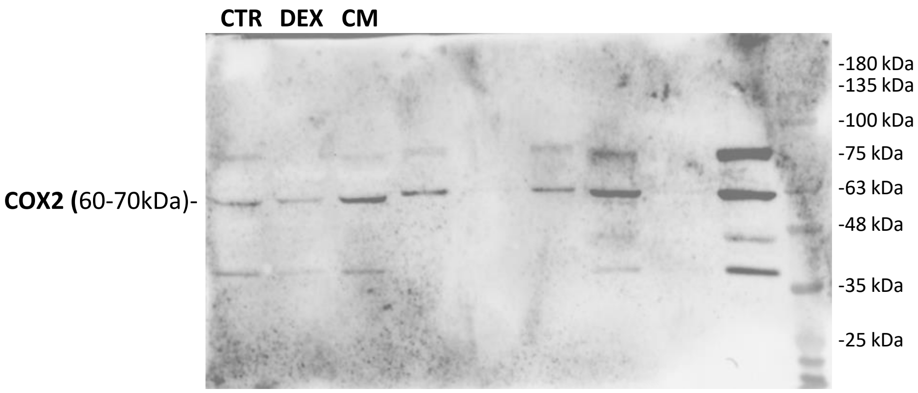

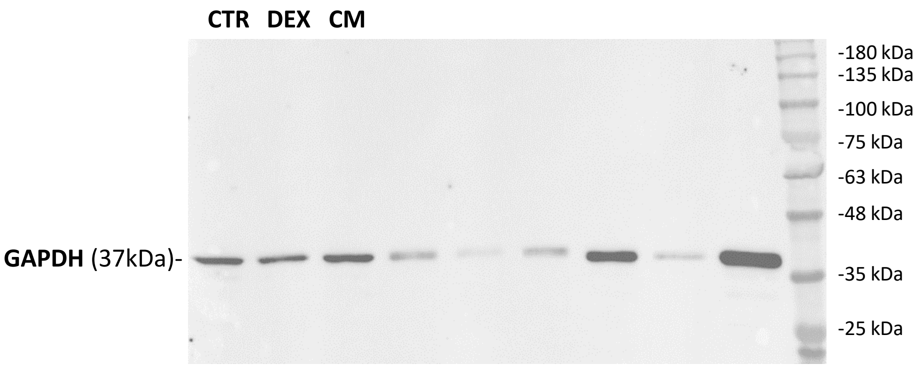

Fig2d and Fig3i

Exp4 and 6

CTR DEX CM CTR DEX CM

COL2A1 (~100-140 kDa)-

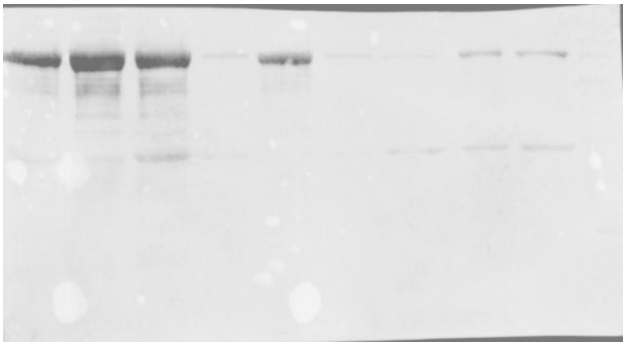

-180 kDa
-135 kDa
-100 kDa
-75 kDa
-63 kDa
-48 kDa
-35 kDa
-25 kDa

CTR DEX CM CTR DEX CM

COX2 (60-70kDa)-

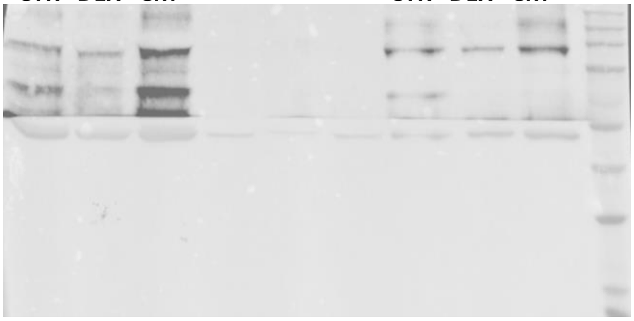

-180 kDa
-135 kDa
-100 kDa
-75 kDa
-63 kDa
-48 kDa
-35 kDa
-25 kDa

CTR DEX CM CTR DEX CM

GAPDH (37kDa)-

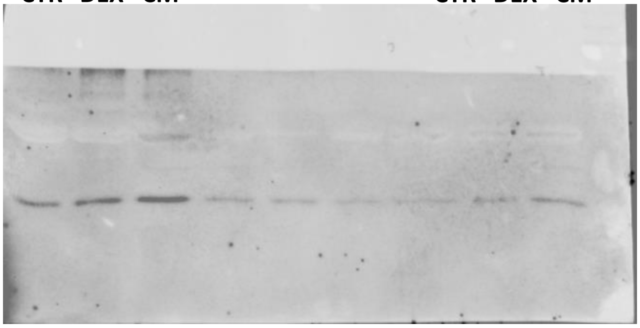

-180 kDa
-135 kDa
-100 kDa
-75 kDa
-63 kDa
-48 kDa
-35 kDa
-25 kDa

Fig2d and Fig3i

Exp7, 8 and 9

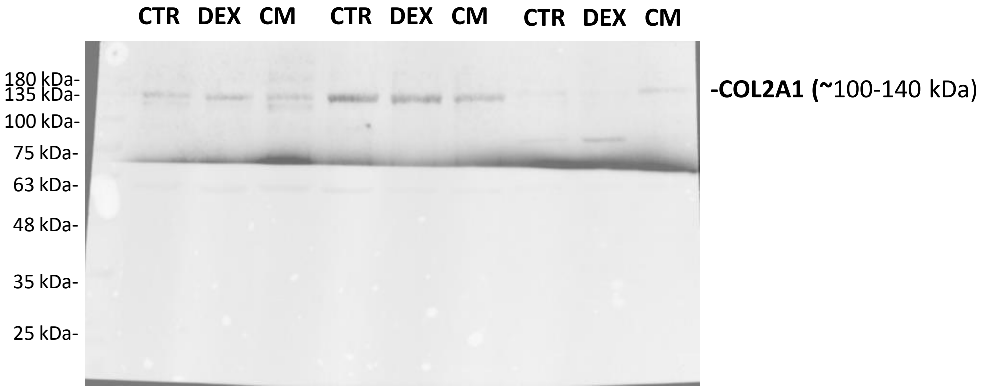

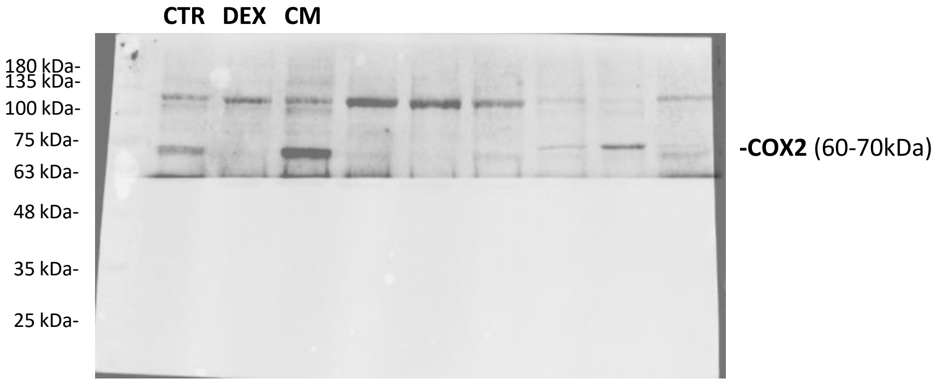

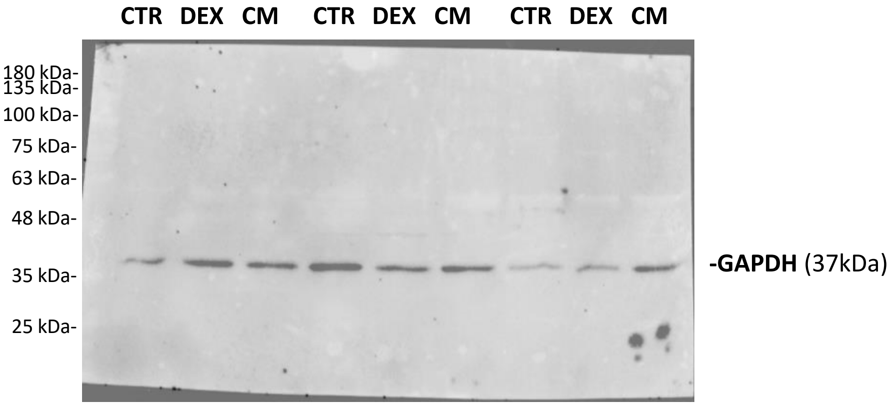

Fig2h and i

Exp6

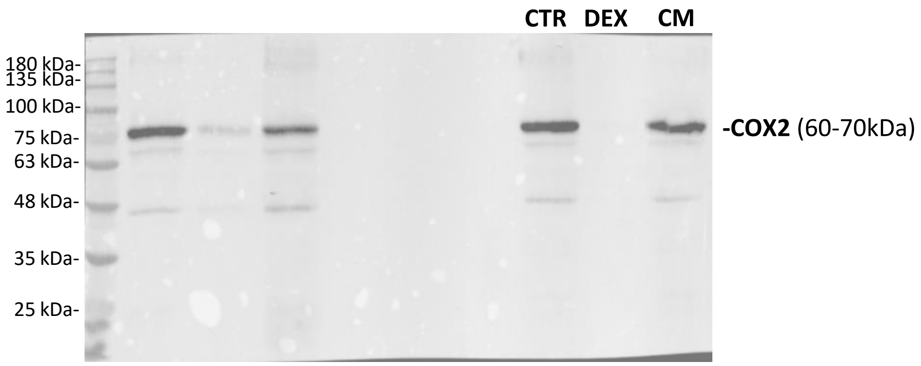

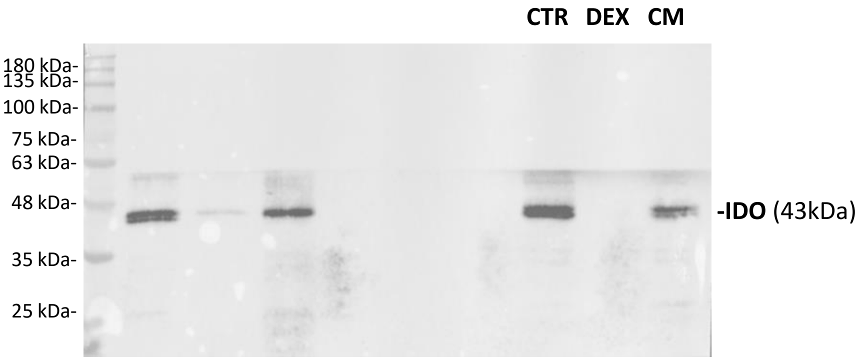

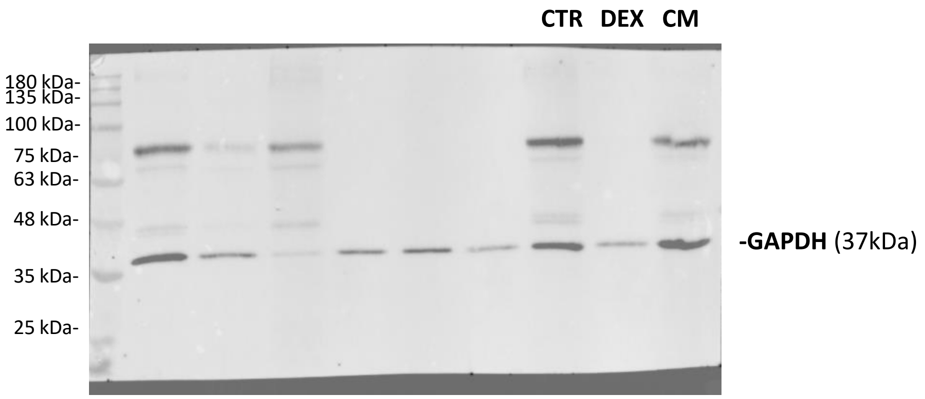

Fig2h and i

Exp7

CTR DEX CM

180 kDa-
135 kDa-
100 kDa-
75 kDa-
63 kDa-
48 kDa-
35 kDa-
25 kDa-

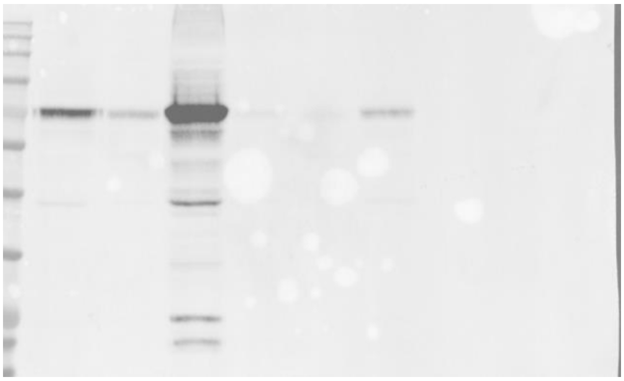

-COX2 (60-70kDa)

CTR DEX CM

180 kDa-
135 kDa-
100 kDa-
75 kDa-
63 kDa-
48 kDa-
35 kDa-
25 kDa-

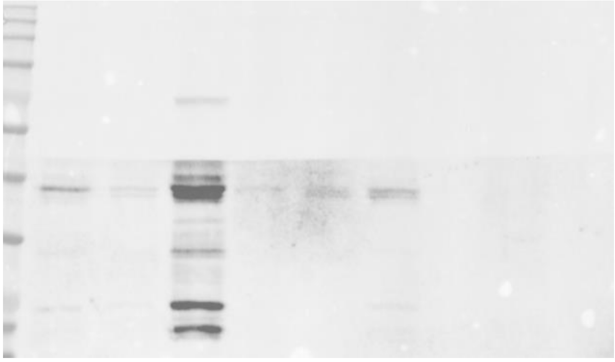

-IDO (43kDa)

CTR DEX CM

180 kDa-
135 kDa-
100 kDa-
75 kDa-
63 kDa-
48 kDa-
35 kDa-
25 kDa-

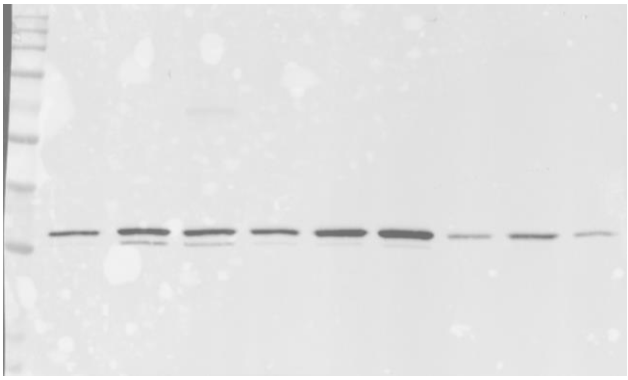

-GAPDH (37kDa)

Fig2h and i

Exp8

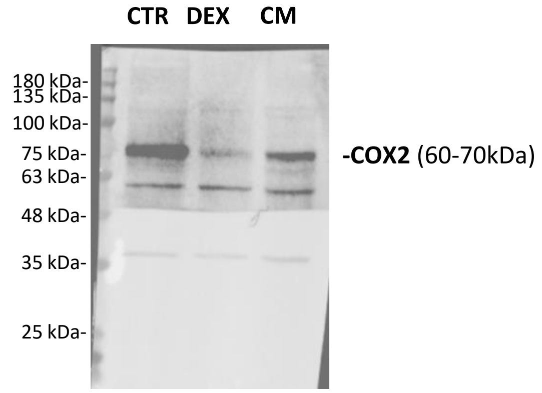

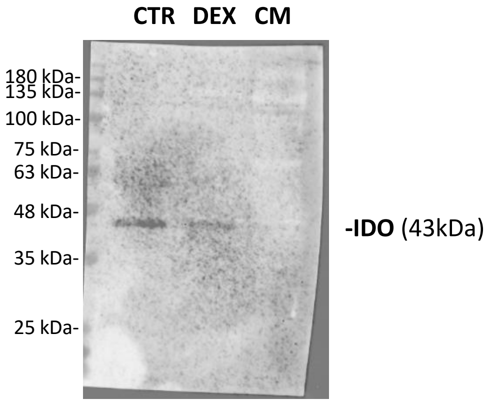

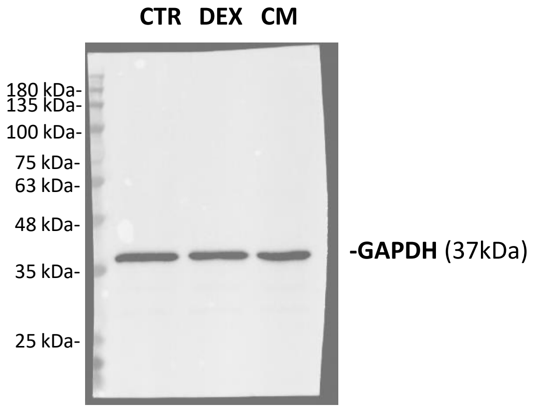

Supplement: Supplementary file 1 [file biology-15-00493-s001.zip › biology-4175071-File S1. WB figures.pdf]
